# Repair of Critical Size Bone Defects Using Synthetic Hydroxyapatite or Xenograft with or without the Bone Marrow Mononuclear Fraction: A Histomorphometric and Immunohistochemical Study in Rat Calvaria

**DOI:** 10.3390/ma14112854

**Published:** 2021-05-26

**Authors:** Jorge Luís da Silva Pires, Jorge José de Carvalho, Mario José dos Santos Pereira, Igor da Silva Brum, Ana Lucia Rosa Nascimento, Paulo Gonçalo Pinto dos Santos, Lucio Frigo, Ricardo Guimaraes Fischer

**Affiliations:** 1 Department of Periodontology, PhD Candidate in Periodontology, School of Dentistry, State University of Rio de Janeiro, Rio de Janeiro 20551-030, Brazil; 2 Laboratory of Cell Ultrastructure and Tissue Biology, Department of Histology and Embryology, State University of Rio de Janeiro, Rio de Janeiro 20550-900, Brazil; jjcarv@gmail.com (J.J.d.C.); mariojsp@gmail.com (M.J.d.S.P.); igor_brum1@hotmail.com (I.d.S.B.); alrosa22@hotmail.com (A.L.R.N.); 3 Department of Periodontology, School of Dentistry, State University of Rio de Janeiro, Rio de Janeiro 20551-030, Brazil; pgps@domain.com.br (P.G.P.d.S.); ricfischer@globo.com (R.G.F.); 4 Department of Periodontology, School of Dentistry, Universidade Guarulhos, Guarulhos 07023-070, Brazil; luciofrigo@uol.com.br; 5 Department of Periodontology, Pontifical Catholic University of Rio de Janeiro, Rio de Janeiro 22451-900, Brazil

**Keywords:** biomaterials, bone marrow mononuclear fraction, bone regeneration, critical size bone defect

## Abstract

Bone defects are a challenging clinical situation, and the development of hydroxyapatite-based biomaterials is a prolific research field that, in addition, can be joined by stem cells and growth factors in order to deal with the problem. This study compares the use of synthetic hydroxyapatite and xenograft, used pure or enriched with bone marrow mononuclear fraction for the regeneration of critical size bone defects in rat calvaria through histomorphometric (Masson’s staining) and immunohistochemical (anti-VEGF, anti-osteopontin) analysis. Forty young adult male rats were divided into five groups (n = 8). Animals were submitted to critical size bone defects (Ø = 8 mm) in the temporoparietal region. In the control group, there was no biomaterial placement in the critical bone defects; in group 1, it was filled with synthetic hydroxyapatite; in group 2, it was filled with xenograft; in group 3, it was filled with synthetic hydroxyapatite, enriched with bone marrow mononuclear fraction (BMMF), and in group 4 it was filled with xenograft, enriched with BMMF. After eight weeks, all groups were euthanized, and histological section images were captured and analyzed. Data analysis showed that in groups 1, 2, 3 and 4 (received biomaterials and biomaterials plus BMMF), a significant enhancement in new bone matrix formation was observed in relation to the control group. However, BMMF-enriched groups did not differ from hydroxyapatite-based biomaterials-only groups. Therefore, in this experimental model, BMMF did not enhance hydroxyapatite-based biomaterials’ potential to induce bone matrix and related mediators.

## 1. Introduction

The regeneration of bone defects represents one of the biggest challenges in implantology. Alveolar bone defects can occur due to several factors, and the physiological bone resorption after extraction with the preservation of the dental alveolus, has been a topic highly addressed in the literature [1,2,3,4,5,6]. Moreover, tooth–facial trauma, periodontal disease, and endodontic treatment failure, in addition to bone/tooth-related cysts and tumors that affect the jaws may cause bone resorption [7,8,9].

The bone grafts commonly used in bone reconstruction surgeries are autogenous, allogeneic, xenogenous bones and alloplastic (synthetic hydroxyapatite (HA) and ß-tricalcium phosphate (ß-TCP)). However, only autogenous bone graft is endowed with osteogenic capacity and is considered the gold standard parameter for comparisons. However, the removal of an autogenous graft often carries a significant risk of morbidity [7].

A promising approach to bone regeneration was established by the identification of multipotent stem cells, such as bone marrow stromal cells. Tissue engineering studies were carried out using bone marrow-derived stem cells, using different types of extraction method: bone marrow aspirate (BMA), bone marrow aspirate concentrate (BMAC), bone marrow mononuclear fraction (BMMF), stem cell culture of origin mesenchymal (MSCs) [5,10,11,12,13,14,15,16,17], and stem cell culture of adipose origin (ASC) [17,18].

Mesenchymal stem cell (MSC) is an undifferentiated cell, which can self-replicate and differentiate into various tissue types, including bone tissue [19]. The development of protocols for clinical use with BMA, BMAC, BMMF, MSCs and ASC was conducted. The main goal was to restore the native cell population without the need to remove large grafts from donor areas.

Stem cell therapy is usually accompanied by different types of scaffolds due to its soluble nature, and hydroxyapatite-based biomaterials are the evident scaffold candidates, when bone tissue is considered. Therapy goals, however, remains to be on a level with the gold standard (autogenous bone) but with less morbidity [7,9,10,16,20,21,22,23,24,25,26,27].

Therefore, the aim of the present work is to provide a comparative study, using histomorphometric and immunohistochemical analysis of two grafting biomaterials, pure and enriched with BMMC. They were used in the regeneration of critical size bone defects in rats’ calvaria to evaluate the possible enhancement of bone matrix production and related mediators.

## 2. Materials and Methods

### 2.1. Experimental Design

This study was approved by the Ethics Committee for the Use of Experimental Animals (CEUA) of the Instituto de Biologia Roberto Alcantara Gomes (IBRAG) under registration #016/2018 and followed the ARRIVE Guidelines.

Forty-eight young adult male rats (40 rats in 5 experimental groups of 8 animals and a further 8 bone marrow donor rats), Sprague Dawley, aged 12 weeks, weighing 350 to 400 g, were kept in the Department of Histology and Embryology (DHE) facility at the State University of Rio de Janeiro (UERJ) under controlled conditions (temperature 21 ± 2 °C, humidity 60 ± 10%, 12 h inverted light cycle—light/dark and air replacement cycle 15 min/h), received standard balanced feed (Quintia/Nuvilab feed, Canguiri, Colombo, Parana, Brazil), and filtered water treated ad libitum, throughout the experiment.

Forty rats were used in five experimental groups of eight animals, assigned as: control group, group 1, group 2, group 3, and group 4. Eight rats were used as bone marrow donors to provide BMMF to experimental groups: group 3 (synthetic hypoxiapatite enriched with BMMF), and the group 4 (xenograft enriched with BMMF).

### 2.2. Obtaining the Bone Marrow Mononuclear Fraction (BMMF)

There are three specific methods for the separation of cell layers from bone marrow or peripheral blood, namely: (1) separation by density gradient; (2) separation based on cell affinity (positive and negative), (3) separation by cell size. Of these three methods, the density gradient is the most used due to its ease of execution, and it was the one used in this work.

The mononuclear cell fraction was processed using Ficoll Histopaque (Sigma-Aldrich, St Louis, MO, USA) in the following sequence of proceedings:

Bone marrow cells were obtained from femurs and tibiae of Sprague Dawley rats, from the bone marrow donor group.

The skin and muscles adjacent to the femur and tibia were gently moved away to prevent as much blood vessel damage as possible in the region. The femurs and tibia were removed and placed in Petri dishes containing PBS for further detailed dissection.

The bone epiphyses were cut, and the bones placed inside a 1000 µL Eppendorf and centrifuged at 461× *g* for 5 min at 4 °C to separate the bone marrow.

The bone marrow was collected and homogenized with PBS. Samples from each animal’s paw were collected and centrifuged at 461× *g* for 5 min at 4 °C. The cell pellet was resuspended in 4 mL of DMEM (Eagle Medium modified by Dulbecco, Sigma-Aldrich, St Louis, MO, USA) without serum, pH 7.2.

After a careful addition of 4 mL of Ficoll (Histopaque 1077, Sigma-Aldrich, St Louis, MO, USA), the tubes were centrifuged at 819× *g* for 30 min at room temperature. After centrifugation, the different layers became clear, previous to layers separation: in the upper phase is the plasma and its soluble constituents, in the interphase the mononuclear cells, just below the Ficoll layer and below the layer containing erythrocytes and granulocytes in the form of a cellular sediment at the bottom of the tube.

**Figure 1:**
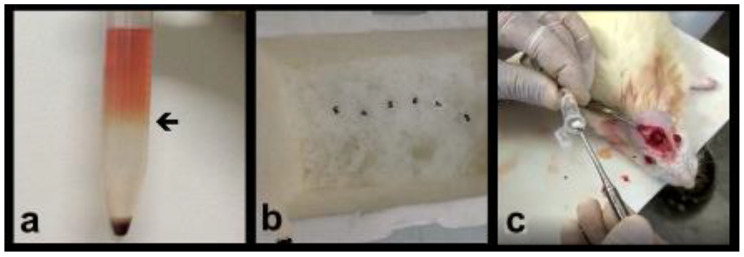
(**a**) Obtaining the BMMF: polypropylene tube after separation by density gradient: plasma platelets, mononuclear cells (arrow), Ficoll-Histopaque, granulocyte–erythrocyte. (**b**) The 1 mL Ependorf tubes were prepared with 1 × 10^6^ cells diluted in 300 μL of ice-cold PBS, pH 7.2. (**c**) The final suspension of the BMMF was added to the synthetic hydroxypatite and the xenograft, 0.10 g in 300 μL of FMMO in the concentration of 1 × 10^6^ cells.

The ring of cells formed at the Ficoll interface, which contained the bone marrow mononuclear cells, was collected and then, the cells were resuspended in 10 mL of PBS pH 7.2 and centrifuged at 461× *g* for 5 min, at 4 °C. (Figure 1a)

The supernatant was discarded, and this process was repeated two more times, for a complete Ficoll removal. Obtained cells were resuspended in 1 mL of sterile cold PBS, pH 7.2, and counted in the Neubauer chamber. Eppendorf tubes were prepared containing 1 × 10^6^ cells diluted in 300 µL of cold PBS, pH 7.2. The final BMMF suspension was added to the synthetic hydroxyapatite and xenograft (groups 3 and 4). (Figure 1b,c)

### 2.3. Bone Grafts Tested in Groups

The five experimental groups of eight animals were subjected to critical bone defects of 8 mm, performed with trephine in the rats’ calvaria and the groups according to the treatment method they were assigned as:

Control group—there was no biomaterial placement in the critical bone defects, only natural clot.

Group 1—the critical bone defects were filled with synthetic hydroxyapatite, 0.10 g of (Alobone poros Osseocon Biomateriais Ltd.a., Rio de Janeiro/RJ, Brazil).

Group 2—the critical bone defects were filled with xenograft, 0.10 g of (Bio-Oss^®^ Small Geistlich Pharma AG, Wolhusen, Switzerland).

Group 3—the critical bone defects were filled with the same synthetic hydroxyapatite used in group 1, with the same weight of 0.10 g (Alobone poros™, Osseocon Biomateriais Ltd.a., Rio de Janeiro/RJ, Brazil), enriched with 300 μL of BMMF 1 × 10^6^, obtained from marrow donor animals. (Figure 1a–c).

Group 4—the critical bone defects were filled with the same xenograft, used in group 2, with the same weight of 0.10 g (Bio-Oss Small™, Geistlich Pharma AG, Wolhusen, Switzerland), enriched with 300 μL of BMMF 1 × 10^6^ obtained from the other four marrow donor animals.

### 2.4. Surgical Protocol and Sample Preparation

Animals were anesthetized with 2% xylazine hydrochloride (Calmiun Agener União Química Farmacêutica Nacional, São Paulo, SP, Brazil), 0.1 mL per 100 g/weight, and ketamine (10 g) (Dopalen-CEVA, São Paulo, SP, Brazil) 0.1 mL per 100 g/weight. After anesthesia, the following surgical sequence procedures were performed: trichotomy of the temporoparietal region with a 15C scalpel blade, antisepsis with Povidine™ (Vic Pharma, São Paulo, SP, Brazil), semilunar incision with full thickness flap with a 15C scalpel blade, detachment of the skin and periosteum, and surgical bone exposure of the temporoparietal region with the Molt detacher (Duflex, Juiz de Fora, MG, Brazil).

Using an 8 mm diameter trephine (Harte instruments, Ribeirão Preto, SP, Brazil) with a reduced speed handpiece 20:1 (Kavo do Brasil, Joinvile, SC, Brazil), coupled to the BLM 600 implant engine (Driller, Carapicuiba, SP, Brazil), the critical bone defects (8 mm) were produced.

**Figure 2:**
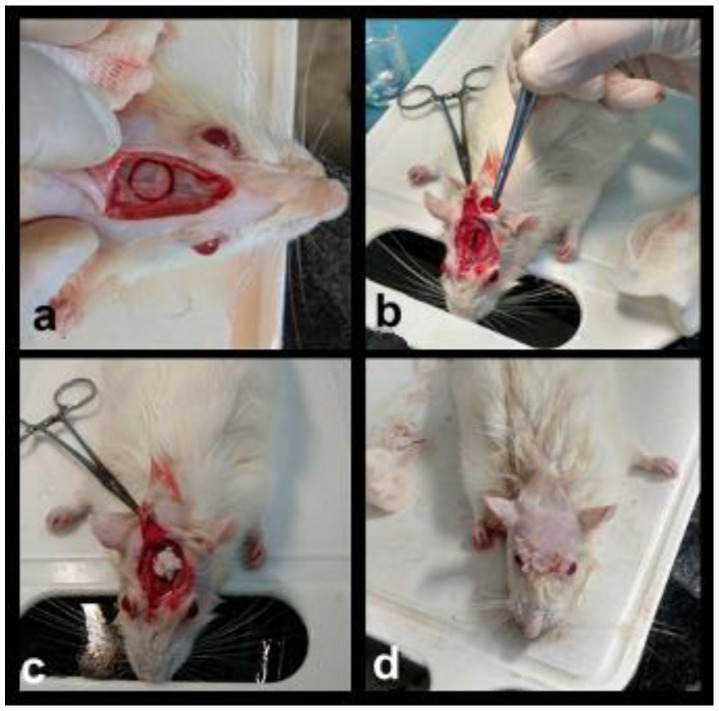
Surgical sequence (**a**) demarcation of the bone defect with 8 mm trephine (**b**) removal of the bone defect with exposure of the dura, (**c**) filling of the bone defect with biomaterial, (**d**) final suture.

After critical bone defects filling, periosteum and skin were repositioned and sutured with resorbable thread, Catgut ™ (Shalon, São Luis de Montes Belos, GO, Brazil), (Figure 2a–d).

The post-anesthetic recovery was monitored clinically, medicated with acetaminophen (paracetamol) (1–2 mg/mL) in the water. The animals were observed, and after completing 8 weeks, all five groups were euthanized through anesthetic overdose of pentobarbital (Cristália,Itapira, SP, Brazil), 180 mg/kg I.P. The skull of each euthanized specimen was decalcified in 10% ethylenediamine-tetra acetic acid (Sigma-Aldrich, St Louis, MO, USA) for 38 days. After decalcification, specimens were included in Paraplast (Sigma-Aldrich, St Louis, MO, USA), and a 7 µm thick microtomy was performed. The histological samples were stained by Masson’s trichrome technique and analyzed by histomorphometry, and by immunostaining techniques for vascular endothelial growth factor (VEGF) and osteopontin (OPN).

### 2.5. Masson Trichromic Staining Protocol

Deparaffinization was achieved by 3 rinses in xilol (Sigma-Aldrich, St Louis, MO, USA) and hydration in decreasing alcohol concentrations baths (100%, 90%, 70%). After a rinse in distilled water, staining with Weigert’s ferric hematoxylin solution was conducted for 10 min. Histological slides were washed in running tap water for 5 min and stained with the scarlet Biebrich (Sigma-Aldrich, St Louis, MO, USA) solution for 5 min. After a rinse in tap water, a phosphotungstic–phosphomolybdic acid (Sigma-Aldrich, St Louis, MO, USA) solution was used as differentiation solution for 10 min. After a further rinse in tap water for 5 min, aniline blue solution was applied on the slides for 5 min. After a bath in 1% glacial acetic acid solution for 3 min, slides were rinsed in tap water again, dehydrated in increasing concentrations of alcohol (70%, 90%, 100%), diaphanized and mounted using Entellan resin (Sigma-Aldrich, St Louis, MO, USA).

### 2.6. Vascular Endothelial Growth Factor (VEGF) and Osteopomtin (OPN) Immunohistochemical Protocol

Histological slides were initially deparaffinized in xylol baths (3 × 5 min) and hydrated in decreasing concentrations of alcohol (100%, 90%, 70%) for 5 min each bath. Slides were incubated in 3% hydrogen peroxide solution for 15 min in the dark, to inhibit endogenous peroxidase. After the inhibition of endogenous peroxidase activity, followed a rinse in PBS buffer pH 7.2 (3 × 5 min)

Antigenic site re-exposure was conducted in citrate buffer solution (pH 6.0 at 96 °C, for 20 min). After the slides cooled and a PBS buffer rinse pH 7.2 (3 × 5 min), nonspecific sites were blocked with PBS/BSA solution (3% for 20 min). After the rinse, slides were incubated with the primary anti-VEGF antibody (Santa Cruz, sc-1876), diluted in PBS/BSA 1% (1:100) and primary anti-OPN antibody (Santa Cruz, sc-21742), diluted in PBS/BSA 1% (1:200) overnight in a refrigerator (4.0 °C) in a humid chamber. After primary antibody incubation, 3 baths of PBS buffer solution pH 7.2 (5 min) were carried out, previously to secondary biotinylated antibody (VECTASTAIN^®^ Universal Quick HRP Kit, Ingold Road, Burlingame, CA, USA) incubation for 1 h, at room temperature. After another PBS buffer solution rinse, slides were incubated with streptavidin (VECTASTAIN^®^ Universal Quick HRP Kit) for 30 min at room temperature. Streptavidin–biotin–peroxidase complex was revealed with diaminobenzidine (DAB) (VECTASTAIN^®^ Universal Quick HRP Kit). Slides were counterstained with hematoxylin solution (0.15%), dehydrated in increased alcohol concentrations (70%, 90%, 100%) (ethanol), diaphanized and mounted using Entellan resin (Sigma-Aldrich, St Louis, MO, USA).

### 2.7. Image Analysis

**Figure 3:**
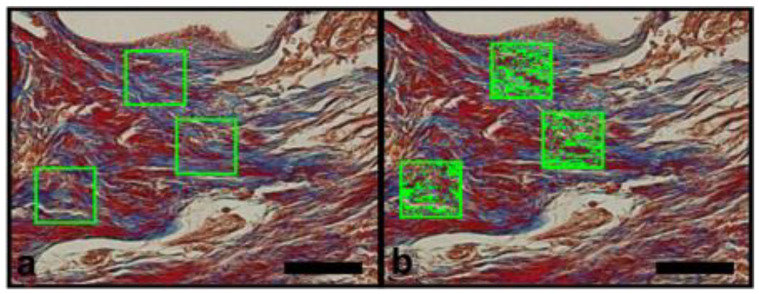
Photomicrographs of histomorphometric analysis, quantification of the area of interest of the sections stained with Masson’s trichrome (×40 magnification) (100 µm scale bar). (**a**) Green square areas limited the quantification sites randomly selected. (**b**) Green dotted area inside green square indicates the Image Pro Plus 7.0 software identification of the bluish staining.

**Figure 4:**
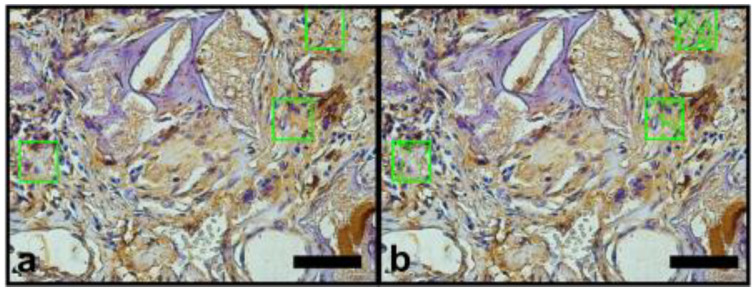
Photomicrographs of immunohistochemistry for VEGF, quantification of the area of interest of the immunostained slides for VEGF (×40 magnification) (100 µm scale bar). (**a**) Green square areas limited the quantification sites randomly selected. (**b**) Green dotted area inside green square indicates the Image Pro Plus 7.0 software identification of the brownish color of the DAB deposits.

**Figure 5:**
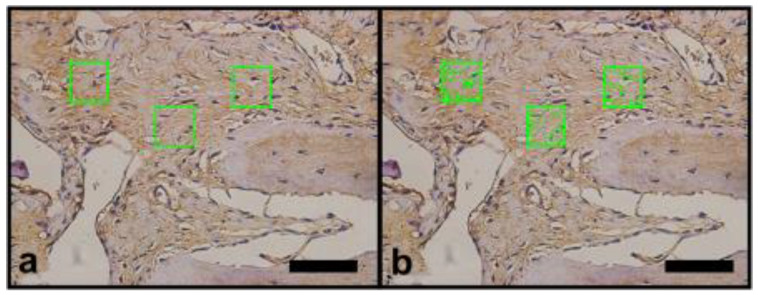
Photomicrographs of immunohistochemistry for osteopontin, quantification of the area of interest of the immunostained slides for OPN (×40 magnification) (100 µm scale bar). (**a**) Green square areas limited the quantification sites randomly selected. (**b**) Green dotted area inside green square indicates the Image Pro Plus 7.0 software identification of the brownish color of the DAB deposits.

The images of the histological sections were captured using the Image Pro Plus 7.0 software (Media Cybernetics, Rockville, MD, USA) coupled to a video microscopy system composed of an Olympus BX-50 microscope and an Olympus DP-72 camera (OLYMPUS Corp., Tokyo, Japan). Previously, the parameters of brightness and white balance were set for ×40 magnification. The images obtained were saved in TIFF format with 12 M pixels, which made the segmentation of the structures of interest more precise. In the histomorphometric analysis, stained by Masson’s trichrome, the blue color in the bone defect indicated the bone matrix in formation, and the red color indicated muscle tissue (Figure 3a,b). Immunostained sites with anti-VEGF or anti-OPN appeared brownish in color and indicated areas related to angiogenesis and presence of bone matrix mineralization activity, respectively (Figure 4a,b and Figure 5a,b).

Each specimen (bone defect), provided by an individual animal was represented by three semi-serial histological sections. Three random microscopic fields were selected but on specific areas: one in the central region two regions in the borders. Each field was analyzed using delimiters of areas of interest, in order to circumvent histological artifacts that could interfere in the quantification of these structures. The segmentation was performed in an interactive way, allowing the correction of bias caused by histological staining techniques. The researcher that conducted the procedure was unaware of the groups tested. The numerical data obtained represented the percentage of area occupied by the structure of interest in the test areas, with the final result of each animal represented by the average of the three cuts (Figure 3b, Figure 4b and Figure 5b).

### 2.8. Statistical Analysis

Data analysis of the histological images stained with Masson’s trichrome, and immunostained for VEGF and OPN were performed with the aid of the Prism 6.0 software (GraphPad Software, Inc., San Diego, CA, USA). The D’Agostino and Pearson omnibus distribution test was performed. For comparison between groups, the Kruskal–Wallis test was used with a significance level of 5% (*p*-value ≤ 0.05) and the Dunn post-test. The comparison of groups 2 to 2 was complemented by the Mann–Whitney test with a significance level of 5% (*p*-value ≤ 0.05), where the type of treatment performed was considered, according to the objectives of work. Pure synthetic hydroxyapatite was compared with xenograft, and that with synthetic hydroxyapatite enriched with BMMF (group 1 was compared with groups 2 and 3); the pure xenograft was compared with the pure synthetic hydroxyapatite and with the xenograft enriched with BMMF (group 2 compared with groups 1 and 4); the synthetic hydroxyapatite enriched with BMMF was compared with the xenograft enriched with BMMF (group 2 compared with group 4), and the control group was compared with all other groups (control group was compared with groups 1,2,3,4).

## 3. Results

### 3.1. General Observations

All animals were observed twice a day in the first 72 h and daily until 14 days. During this period, no signs of pain behavior, bleeding or visible edema was observed. From the second day on, the complete animal behavior (feeding, drinking, grooming) was reestablished. No animal was lost until euthanasia day.

### 3.2. Histomorphometric Results

**Figure 6:**
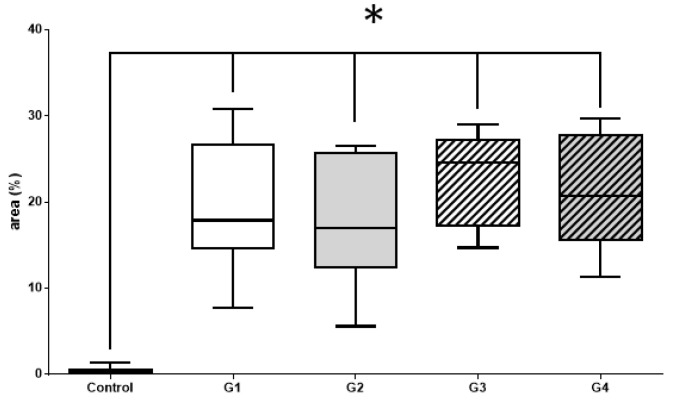
Histomorphometric analysis—Masson’s trichrome-Kruskal–Wallis statistical test with Dunn’s posttest. There was no significant statistical difference between the treated groups, but there was a statistically significant difference between the treated groups and the control group, which did not receive any type of treatment.

**Figure 7:**
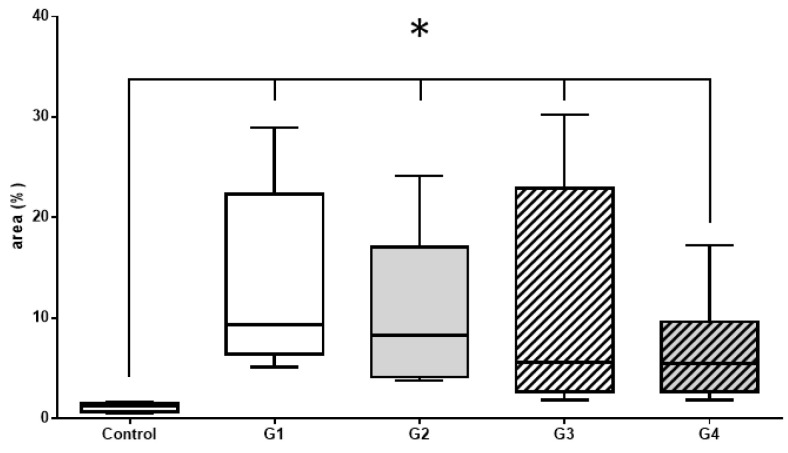
Immunohistochemistry—analysis of results immunomarked sections with VEGF by analyzing the Kruskal–Wallis statistical test with Dunn’s posttest. There was no significant statistical difference between the treated groups, but there was a statistically significant difference between the treated groups and the control group, which did not receive any type of treatment.

**Figure 8:**
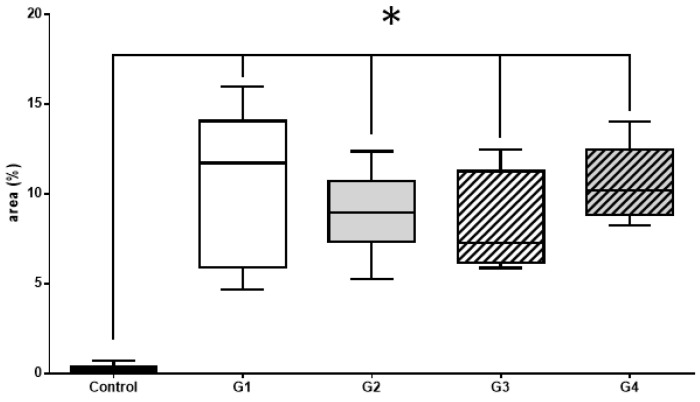
Immunohistochemistry—analysis of results immunomarked sections with OPN by analyzing the Kruskal–Wallis statistical test with Dunn’s posttest. There was no significant statistical difference between the treated groups, but there was a statistically significant difference between the treated groups and the control group, which did not receive any type of treatment.

Histomorphometric and immunohistochemical evaluations with the Kruskal–Wallis test and Dunn’s posttest, showed no significant difference between groups 1, 2, 3, and 4. However, there was a significant difference between the control group and the other groups (1, 2, 3, and 4) (Figure 6, Figure 7 and Figure 8).

The Mann–Whitney test complemented the analysis, comparing in pairs, considering the treatment modality performed, for the respective histomorphometric (Masson’s trichrome) and immunohistochemistry (VEGF and OPN) (Table 1, Table 2 and Table 3).

Hence, BMMF did not enhance the hydroxyapatite-based biomaterials’ potential to promote matrix production, nor stimulated the VEGF and OPN production.

#### 3.2.1. Histomorphometric Masson’s Trichrome Results

Masson’s trichrome staining is a traditional staining technic that is composed of aniline blue and has a strong affinity to basic proteins, including collagen type I, the main organic content of the bone matrix (Figure 6, Table 1).

#### 3.2.2. Histomorphometric VEGF Results

Vascular endothelial growth factor (VEGF) is mitotic-inducing cell modulator, mainly related to promoting blood vessels sprouting and enhancing blood flow in the region. Vascularization is considered a step-limiting event in bone matrix production (Figure 7, Table 2).

#### 3.2.3. Histomorphometric OPN Results

OPN is a highly phosphorylated non-collagenous sialoprotein expressed in all bone cells and it is considered to play an important role in bone formation and resorption (Figure 8, Table 3).

## 4. Discussion

The physical–chemical characteristics of biomaterials, such as porosity, crystallinity, and particle size, directly influence the in vivo biological behavior of biomaterials after their use [8,28,29].

Hydroxyapatite has good cell conductivity and allows a good structure for the fibrin network [30,31]. These characteristics make hydroxyapatite synthetic and bovine clinical alternatives to autogenous bone graft, used in bone graft surgeries with excellent biological responses [30,32,33]. Hydroxyapatite is used in guided bone reconstruction, along with occlusive barriers, titanium mesh, collagen membranes, among other applications [34]. Cortical perforation of the recipient bone, in addition to synthetic bone substitutes, can improve angiogenesis and increase the amount of newly formed bone, especially in the early stages of bone regeneration [35].

Comparing the regeneration of critical bone defects in the calvaria of Sprague Dawley rats, using xenograft and synthetic hydroxyapatite, both pure, demonstrated that there was no statistical difference between them in bone neoformation [36]. The same was observed in our study, where the result of group 1 regenerated with synthetic hydroxyapatite, (Alobone poros™) and group 2 regenerated with Bio-Oss small™ (bovine xenograft) showed no statistically significant difference in histomorphometric evaluation (Masson’s trichrome) and immunostaining for (VEGF and OPN), as observed in the study in rabbit, in the dimensional alterations of the alveolar ridge that occurred following tooth extraction, showing similar tissue responses for the two biomaterials those were placed in the fresh extraction socket [37]. These results were also observed in the randomized clinical study (RTC) with split mouth design in humans, comparing a pure sintered nanohydroxyapatite (NHA) and inorganic bovine bone (ABB), where there was no statistically significant difference [38].

In the study on rabbit calvaria comparing pure hydroxyapatite and pure xenogenous bone and associated with rhBMP-2, both bone replacement materials (HA/SiO and DBBM) showed a similar amount of bone formation over 8 weeks, with the main difference being the addition of rhBMP-2, which may offer additional benefits in terms of newly formed bone. Another difference is that HA/SiO appears to degrade more quickly with a higher turnover rate, leaving room for a little more bone formation, while DBBM appears to degrade at a slower rate [39]. Other studies corroborate the same results in relation to the higher percentage of residual material from the xenogenous graft [40,41].

Among cell therapy techniques, such as bone marrow aspirate (BMA), bone marrow concentrate aspirate (BMAC), bone marrow mononuclear fraction concentrate (BMMF) and bone marrow mesenchymal stem cell culture, the simplest to perform is bone marrow aspirate [42,43], but it seems to fall short of techniques that involve some type of bone marrow processing [7,8,9,10].

Comparing methods, such as obtaining mononuclear fraction using the Ficoll–Histopaque method, and concentrated bone marrow aspirate using the BMAC method, associated with a bovine biomaterial, the difference between the groups was not statistically significant, suggesting that the BMAC system is effective, and a more practical method for clinical application than Ficoll [41]. Similar results were observed in the split mouth study, in which maxillary sinus lift augmentation using the BMAC method was compared with the conventional method, which involves mixing biomaterials with autologous bone [44]. However, in a bone regeneration study carried out with bilateral critical bone defects, it was found that the use of the mononuclear fraction of the bone marrow BMMF associated with the xenogenous biomaterial showed a positive result in the newly formed bone percentage, when compared to the biomaterial alone, and presented approximately half of it, bone formation verified in the autogenous bone [10], being compared to the osteogenic potential of the mesenchymal stem cells derived from the purified marrow, although the BMMF initially contains far fewer progenitor cells of mesenchymal origin. These results suggest new approaches for the treatment of bone defects [45].

In our work, we used BMMF in group 3, where the synthetic hydroxyapatite was enriched with BMMF, and in group 4, where the bovine xenograft was also enriched with BMMF. Both groups showed no statistically significant difference in histomorphometric evaluation (of sections stained with Masson’s trichrome) and immunomarking for VEGF and OPN, either between them, or when compared with the use of pure biomaterials, as in group 1, synthetic hydroxyapatite, and in group 2, bovine xenograft. Hence, the null hypothesis has been accepted.

There is a search for consensus regarding the best methodology for the use of MSCs (mesenchymal stem cells). Although cell cultures can increase the number of osteogenic cells, osteogenic potential was not observed when using the cell culture technique in comparison to fresh bone marrow [46,47]. The culture of stem cells has some disadvantages. Compared to the mononuclear fraction or fresh bone marrow, they require costs and time between harvest and transplantation, there is a risk of contamination and a lack of agreement regarding the number of cells needed [48].

Although bone regeneration based on tissue engineering using mesenchymal stem cells has a solid scientific knowledge, choosing between tissue engineering, using mesenchymal stem cells associated with a biomaterial, or using the biomaterial alone, must be based on scientific evidence [49].

## 5. Conclusions

With the limitation of this study, both the synthetic hydroxyapatite and xenograft enriched with bone marrow mononuclear fraction were not demonstrated to influence the regeneration of critical size bone defects when compared to the use of these biomaterials alone. However, more studies should be carried out to confirm these results.

## Figures and Tables

**Table 1:** Histomorphometric analysis—Masson’s trichrome using the Mann–Whitney statistical test with a significance level of 5% (*p* ≤ 0.05).

Masson’s Trichrome Histomorphometry—Mann–Whitney Test	*p* Value
Control vs. G1	<0.001
Control vs. G2	<0.001
Control vs. G3	<0.001
Control vs. G4	<0.001
G1 vs. G2	0.6294
G1 vs. G3	0.3231
G2 vs. G4	0.4945
G3 vs. G4	0.7023

**Table 2:** Immunohistochemistry analysis of results immunomarked sections with VEGF—through the statistical comparison test through the Mann–Whitney test with a significance level of 5% (*p* ≤ 0.05).

VEGF—Mann–Whitney Test	*p* Value
Control vs. G1	<0.001
Control vs. G2	<0.001
Control vs. G3	<0.001
Control vs. G4	<0.001
G1 vs. G2	0.3754
G1 vs. G3	0.1930
G2 vs. G4	0.4331
G3 vs. G4	0.8541

**Table 3:** Immunohistochemistry—analysis of results immunomarked sections with OPN—using the statistical comparison test using the Mann–Whitney test with a significance level of 5% (*p* ≤ 0.05).

Osteopontin—Mann–Whitney Test	*p* Value
Control vs. G1	<0.001
Control vs. G2	<0.001
Control vs. G3	<0.001
Control vs. G4	<0.001
G1 vs. G2	0.4945
G1 vs. G3	0.4331
G2 vs. G4	0.2317
G3 vs. G4	0.1593
